# Exploring the cancer methylome

**DOI:** 10.1186/1753-6561-6-S6-O24

**Published:** 2012-10-01

**Authors:** Benjamin P Berman, Daniel J Weisenberger, Toshinori Hinoue, Houtan Noushmehr, Yaping Liu, Joseph F Aman, Toshinori Hinoue, Hui Shen, Simeen Malik, Swapna Mahurkar, Timothy Triche, Zachary Ramjan, Charles M Nicolet, David Van Den Berg, Leslie Cope, James G Herman, Stephen B Baylin, Peter W Laird

**Affiliations:** 1 USC Epigenome Center, University of Southern California, Keck School of Medicine, Los Angeles, CA, USA; 2 Johns Hopkins School of Medicine, Baltimore, MD, USA

## 

Cancer develops not only as a result of genetic mutations and genomic rearrangements, but also as a consequence of numerous epigenetic alterations, including extensive changes in the distribution of DNA methylation throughout the genome. DNA methylation changes contribute directly to cancer by transcriptional silencing of tumor-suppressor genes through promoter CpG island hypermethylation. Broad epigenomic analysis of human tumors can reveal relationships between large numbers of epigenetic events and can provide insight into the mechanisms underlying concerted epigenetic change. Genomic loci targeted by Polycomb Group Repressors in embryonic stem cells, and involved in cellular differentiation, are predisposed to aberrant DNA methylation in cancer cells, suggesting that an epigenetic block to cellular differentiation may sometimes be an initiating event in carcinogenesis. The very strong associations between distinct epigenetic subtypes, such as CpG Island Methylator Phenotypes (CIMP) and specific somatic genetic events, such as *BRAF* mutation in colorectal cancer and *IDH1* mutation in glioblastoma multiforme are consistent with an early role for DNA methylation alterations, providing a favorable cellular context for the subsequent somatic mutation. The analysis of whole methylomes at single-basepair resolution reveals that cancer-associated changes occur differentially across defined regions of the genome associated with the nuclear lamina. It is apparent that epigenomic analysis is essential for a full understanding of the relationship between alterations in the cancer genome and the origin and clinical diversity of individual tumors.

